# The Potential of Food Protein-Derived Bioactive Peptides against Chronic Intestinal Inflammation

**DOI:** 10.1155/2020/6817156

**Published:** 2020-09-09

**Authors:** Wanying Zhu, Liying Ren, Li Zhang, Qinqin Qiao, Muhammad Zahid Farooq, Qingbiao Xu

**Affiliations:** ^1^Shanxian Central Hospital, Heze 274300, China; ^2^College of Information Engineering, Fuyang Normal University, Fuyang 236000, China; ^3^College of Animal Sciences and Technology, Huazhong Agricultural University, Wuhan 430070, China; ^4^State Key Laboratory of Animal Nutrition, Institute of Animal Sciences, Chinese Academy of Agricultural Sciences, Beijing 100193, China

## Abstract

Inflammation can cause various chronic diseases like inflammatory bowel diseases. Various food protein-derived bioactive peptides (BAPs) with anti-inflammatory activity have the potential to manage these diseases. The aim of this paper is to overview the mechanisms and the molecular targets of BAPs to exert anti-inflammatory activity. In this review, the *in vitro* and *in vivo* effects of BAPs on intestinal inflammation are highlighted. The mechanism, pathways, and future perspectives of BAPs as the potential sources of therapeutic treatments to alleviate intestinal inflammation are provided, including nuclear factor-*κ*B, mitogen-activated protein kinase, Janus kinase-signal transducer and activator of transcription, and peptide transporter 1 (PepT1), finding that PepT1 and gut microbiota are the promising targets for BAPs to alleviate the intestinal inflammation. This review provides a comprehensive understanding of the role of dietary BAPs in attenuating inflammation and gives a novel direction in nutraceuticals for people or animals with intestinal inflammation.

## 1. Introduction

Inflammation is a normal immune defense that is generated from the immune system responding to pathogen and infection. Inflammation can cause various chronic diseases, such as inflammatory bowel diseases (IBD), asthma, cancer, cardiovascular diseases, obesity, and diabetes [1]. The intestinal mucosa can be damaged by IBD with chronic inflammatory disorders, including ulcerative colitis (UC) and Crohn's disease (CD). UC is an inflammation of the colon mucosa and submucosa continuity affecting the rectal area, while CD is a full-thickness inflammation discontinuity affecting the terminal ileum and colon or anus [2]. Until now, the aetiology of intestinal inflammation and IBD remains unclear.

In the intestines of human and animals, dietary proteins are digested into free amino acids and peptides by enzymatic hydrolysis. Some peptides consisting of 2–20 AAs with biological function are named bioactive peptides (BAPs), such as anti-inflammation, antihypertension, antioxidation, antidiabetics, anticancer, antimicrobics, antiadhesion, dipeptidyl peptidase IV inhibition, opioid, and immunomodulation [3]. Conventional drug treatments have adverse side effects, such as potential toxicity and immunogenicity [4]. In recent years, BAPs have attracted more and more attention to treat chronic inflammation diseases as a result of their safety [1, 5].

However, limited information of the anti-inflammatory mechanisms of the action of these BAPs is available. In this review, food protein-derived BAPs against intestinal inflammation *in vitro* and *in vivo* are discussed. Their molecular targets and the action pathways are overviewed and highlighted. Understanding of the anti-inflammatory actions of BAPs can facilitate further research on managing chronic intestinal inflammation and diseases. Therefore, the purpose of this paper is to highlight the roles of BAPs in anti-inflammatory activity and provide future perspectives for the application of BAPs as potential sources of therapeutic management of chronic intestinal diseases.

## 2. Intestinal Inflammation

Inflammation can activate protective proinflammatory mediators, such as interleukin- (IL-) 1, IL-6, IL-8, IL-12, interferon-*γ* (INF-*γ*), and tumor necrosis factor-*α* (TNF-*α*) in immune responses, which include T and B lymphocytes. The activated B lymphocytes can produce antibodies, such as IgA, IgG, IgM, and IgE. The T lymphocyte cells consist of CD4^+^ and CD8^+^ T cells. CD4^+^ T cells, named helper T lymphocytes (Th), have immune regulatory function by secreting cytokines, being classified into Th1 and Th2. Th1 can release IL-2, IFN-*γ*, and TNF-*α* to promote cellular immunological response, whereas Th2 can release IL-4 and IL-10 to improve immunoresponse, while CD8^+^ T cells have the function to kill the target cells [6].

Progression of inflammation has four steps: inducers, pathways, mediators, and inflammatory response [7]. The inducers (LPS, dextran sodium sulfate (DSS), 2,4,6-trinitrobenzene sulfonic acid (TNBS), or toxicant) stimulate the sensors that can activate pathways, including nuclear factor-*κ*B (NF-*κ*B) and mitogen-activated protein kinase (MAPK). Then, inflammatory mediators (IL-8, TNF-*α*, monocyte chemoattractant protein-1 (MCP-1), or reactive oxygen species (ROS)) are released, leading to the inflammatory response [7]. Proinflammatory cytokines produced mainly by macrophages and mast cells lead to inflammation, while anti-inflammatory cytokines, such as IL-4, IL-10, and transforming growth factor *β* (TGF-*β*), reduce the production of procytokines in macrophage cells as agonists of toll-like receptor [7]. In *in vivo* studies, TNBS and DSS are commonly used to induce intestinal inflammation in animal models, causing immune alterations, gut physiology and morphology changes, and colitis symptoms [8]. Moreover, administration of DSS can lead to higher intraluminal IgG [9]. In UC patients, IgG production is dramatically high in the gut; therefore, IgG is an index to grade IBD. Thus, these cytokines with pathology may be the targets for BAPs to prevent chronic inflammation. In addition, it is also known that oxidative stress is associated with chronic intestinal inflammation, and it can decrease antioxidant defenses in the colonic mucosa. Additionally, ROS are released from immune cells and can be overwhelmed by oxidative stress. Therefore, antioxidative BAPs are the candidates for antioxidant defense in inflammatory gut [10], such as IRW [11], IQW [12], EAMAPK, and AVPYPQ [13]. Soybean-derived lunasin can also enhance antioxidant defenses and inhibit inflammation [14, 15].

## 3. Anti-Inflammatory Peptides Derived from Food Proteins

**Figure 1:**
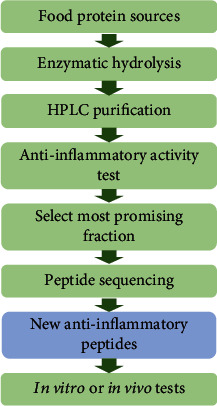
Classical steps toward the production and purification of anti-inflammatory peptides from food protein sources. HPLC: high-performance liquid chromatography. This figure was adapted from previous reports [3, 48, 81].

In the gut of human or animals, the BAPs encrypted in parent proteins can be released by various enzymatic digestion. However, there are several classical steps toward the *in vitro* production of novel BAPs from various food protein sources: enzymatic hydrolysis, purification by high-performance liquid chromatography, selection of most promising fraction, peptide sequencing, and final *in vitro* or *in vivo* bioactivity test (Figure 1) [5, 7, 16]. Due to their safety, the anti-inflammation potential of food-derived BAPs has become an active research area, and the intestinal tract is a main target of BAPs.

Recent knowledge of anti-inflammatory BAPs in *in vitro* studies with a concentration of 20-1000 *μ*M was evaluated using mammalian cells induced by TNF-*α*, LPS, or H_2_O_2_, such as murine RAW 264.7 macrophages and human intestinal epithelial cell line Caco-2 cells (Table 1). There are many food-derived BAPs that can inhibit inflammation via the MAPK or NF-*κ*B pathway (Table 1), such as CR, FL, HC, LL, MK [17], DEDTQAMPFR, DEDTQAMPF [18], DYKKY [19], EAMAPK, AVPYPQ [13], FLV [20], GPETAFLR [21], GPR [22], IPAV [23], IRW [24], IQW [12], LDAVNR, MMLDF [25], MLGATSL, MSYSAGF [18], PAY [26], PRRTRMMNGGR, MGPAMMRTMPG [27], QCQQAVQSAV [28], QQQQQGGSQSQ, QEPQESQQ, QQQQQGGSQSQSQKG, PETMQQQQQQ [29], SSEDIKE [30], VPP [31], IPP [32], VPY [33], VH, LAN, IA, AL [34], *β*-Ala-His [35], and pyroGlu-Leu [36]. Egg ovotransferrin-derived tripeptide IRW exhibits the anti-inflammatory effect through the NF-*κ*B pathway by inhibiting p65 and p50 [24]. Moreover, whey protein-derived tetrapeptide IPAV can reduce IL-8 production via the NF-*κ*B and MAPK pathways [23]. While BAPs have shown potential as anti-inflammatory agents in cultured cells, further *in vivo* studies and underlying mechanism are still necessary to verify their effectiveness in managing chronic inflammation [2].

## 4. Pathways Involved in the Inhibition of Chronic Intestinal Inflammation by BAPs

**Figure 2:**
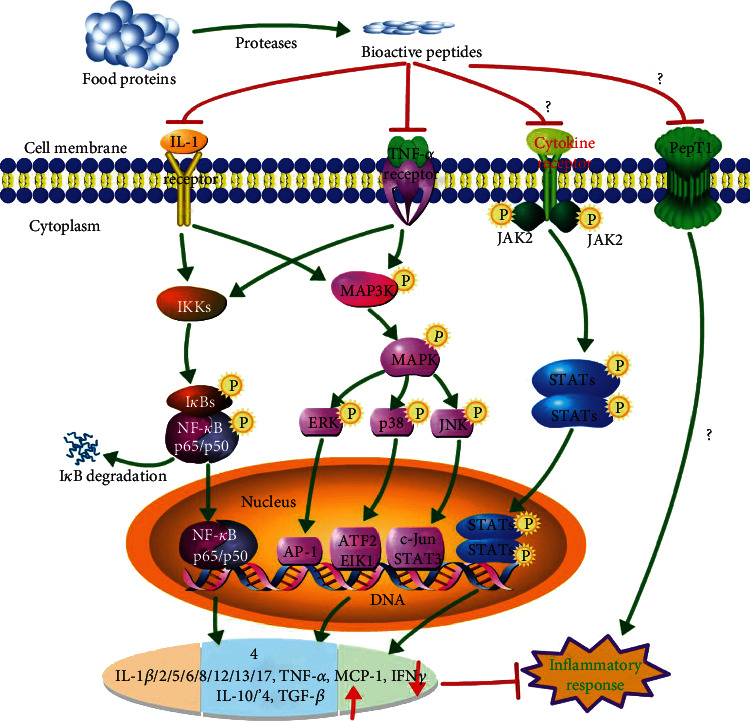
Schematic diagram of possible anti-inflammatory mechanism of bioactive peptides derived from food proteins. The anti-inflammatory activity may be via the following four pathways: NF-*κ*B, MAPK, JAK-STAT, and PepT1. IL-1: interleukin-1; LPS: lipopolysaccharides; MAPK: mitogen-activated protein kinase; MAP3K: MAPK kinase kinase; NF-*κ*B: nuclear factor-kappa B; TGF-*β*: transforming growth factor *β*; TNF-*α*: tumor necrosis factor *α*; JAK-STAT: Janus kinase-signal transducer and activator of transcription. This diagram was drawn using an online pathway builder tool (http://www.proteinlounge.com). Adapted from previous reports [2, 7, 10, 20, 37–41].

There are four possible mechanism pathways for BAPs to attenuate chronic intestinal inflammation: NF-*κ*B, MAPK, Janus kinase-signal transducer and activator of transcription (JAK-STAT), and peptide transporter 1 (PepT1) (Figure 2) [2, 7, 10, 20, 37–41]. Through inhibiting these pathways, BAPs can act the anti-inflammatory function in intestinal cells.

Among these pathways, the NF-*κ*B and MAPK pathways are two main pathways for BAPs to inhibit inflammation [7]. The NF-*κ*B is a key regulator of the expression and secretion of inflammatory cytokines (TNF-*α*, IL-1*β*, IL-6, and IL-8) and also plays a vital role in the expressions of cyclooxygenase-2 (COX-2) and inducible nitric oxide synthase (iNOS) [42]. Inflammatory stimuli (IL-1*β*, LPS, TNF-*α*, viruses, or oxidative stress) activate inhibitory *κ*B kinases (IKK*α*, IKK*β*, and IKK*γ*), leading to phosphorylation of a potential cytoplasmic transcription factor that contains an inhibitor of *κ*B (I*κ*B*α*, I*κ*B*β*, and I*κ*B*γ*) and I*κ*B*α* degradation [42]. NF-*κ*B is a family of transcription factor proteins, including five subunits: p65 (RelA), p50, p52, Rel, and RelB. After dimer p65/p50 is released into the cytosol, it can be translocated into the nucleus and initiates target gene transcription for proinflammatory factors, causing inflammation (Figure 2) [2, 42]. Many food-derived BAPs can inhibit inflammation via this NF-*κ*B pathway, such as DYKKY [19], GPR [22], IRW [24], IQW [12], MLGATSL, MSYSAGF [18], pyroGlu-Leu [36], and TMKLLLVTL [43].

Another major signaling pathway, MAPK, can regulate many cellular activities, including proliferation, differentiation, death, and immune response. The stimulus and MAP3K phosphorylation can mediate the phosphorylation of the downstream MAP2K and MAPK, which contain three subfamilies: p38, extracellular signal-regulated kinases (ERK1 and ERK2), and c-Jun N-terminal kinase (JNK). In unstimulated cells, JNK mainly exists in the cytoplasm, but there is also some distribution in the nucleus. After being stimulated, JNK accumulates in the nucleus and causes the corresponding gene (IL-1 and TNF-*α*) expression, resulting in inflammatory response (Figure 2) [44]. Various food protein-derived BAPs can inhibit inflammation via this MAPK pathway, such as DEDTQAMPFR, DEDTQAMPF [18], FLV [20], MLGATSL, MSYSAGF [18], *β*-Ala-His [35], pyroGlu-Leu [36], DIKTNKPVIF [45], VPP [46], WH [41], *γ*-EC, and *γ*-EV [47].

Along with the above two pathways, the JAK-STAT pathway is also important for inflammatory response and can regulate hematopoietic cell development and inflammatory cytokines. Phosphorylation of JAK and STATs can form the dimer translocated to the nucleus [38]. BAPs can attenuate inflammation by inhibiting phosphorylation of JAK and STATs. However, the role of this pathway needs further verification for the anti-inflammation of BAPs. The translocations and activation of the substrate proteins from the above three pathways, including transcription factors in the nucleus (AP-1, ATF-2, EIK1, and c-Jun), cause the change of target genes, reducing the productions of proinflammatory cytokines, including IL-1*β*, IL-2, IL-5, IL-8, IL-12, IL-13, IL-17, TNF-*α*, MCP-1, and IFN-*γ*. The overexpression of these proinflammatory mediators and the downexpression of anti-inflammatory cytokines (IL-4, IL-10, and TGF-*β*) can lead to intestinal inflammation. Through regulating these pathways and cytokines, BAPs can attenuate chronic intestinal inflammation and diseases.

## 5. Mechanism of Food-Derived Anti-Inflammatory Peptides to Exert Bioactivities

**Figure 3:**
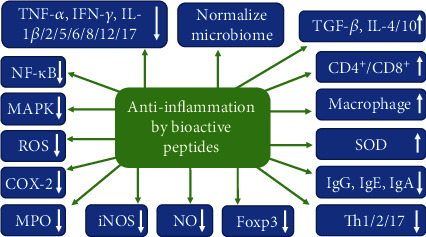
The potential mechanisms of anti-inflammatory action of food-derived bioactive peptides. CD4^+^/CD8^+^: splenic T lymphocyte subpopulations; COX-2: cyclooxygenase-2; Foxp3: a transcription factor that drives treg phenotypic differentiation; iNOS: inducible oxide nitric synthase; IFN-*γ*: interferon-*γ*; IL-1*β*: interleukin-1*β*; MAPK: mitogen-activated protein kinase; MPO: myeloperoxidase; NF-*κ*B: nuclear factor-*κ*B; NO: nitric oxide; ROS: reactive oxygen species; SOD: superoxide dismutase; TNF-*α*: tumor necrosis factor *α*; TGF-*β*: transforming growth factor *β*; Th1/2/17: T-helper-cell-associated cytokine 1/2/17. This figure was adapted from previous reports [7, 48].

The potential anti-inflammatory mechanisms of BAPs derived from food proteins through regulating various cytokines or systems are shown in Figure 3 [7, 48]. The secretions and expressions of proinflammatory cytokines IL-1*β*, IL-2, IL-5, IL-6, IL-8, IL-12, IL-17, TNF-*α*, and IFN-*γ* can be inhibited by BAPs, as well as the activations of NF-*κ*B and MAPK pathways, COX-2, ROS, iNOS, and nitric oxide (NO). ROS are associated with inflammatory diseases, and NO is synthesized by NO synthase (NOS) enzyme (iNOS), and the inhibition of iNOS and ROS activities can suppress NO production. BAPs can also inhibit the expression and release of a transcription factor that drives treg phenotypic differentiation (Foxp3) and T-helper-cell-associated cytokines (Th1, Th2, and Th17) and the secretions of IgG, IgE, and IgA. On the other side, secretions and expressions of anti-inflammatory cytokines (IL-4, IL-10, and TGF-*β*), CD4^+^/CD8^+^, numbers of macrophages, and superoxide dismutase (SOD) activity can be increased by BAPs. In addition, the gut microbiome, which is an active topic in health, can be normalized by BAPs [7, 48]. In conclusion, these cytokines and pathways are the molecular targets and mechanisms for BAPs to regulate the intestinal inflammation of human and animals.

Milk-derived VPP and IPP can exhibit beneficial effect in an animal colitis model through anti-inflammatory action for these targets [49]. VPP also reduced TNF-*α* and IL-1*β* expression and macrophage accumulation and activation, inhibited adipose inflammation in mice via angiotensin-converting enzyme-dependent cascade [31], and moderated monocyte adhesion to inflamed endothelia via the MAPK-JNK pathway [50]. In addition, tripeptides IRW and IQW downregulated the expression of inflammatory proteins via the NF-*κ*B pathway [12, 24]. Generally, these BAPs can inhibit the expression of cytokines and mediate the NF-*κ*B and MAPK pathways [1].

## 6. The *In Vivo* Studies of BAPs on Inflammation

For the *in vivo* studies of BAPs, various inflammatory models have been used, typically colitis in mice induced by DSS and TNBS. As observed in human CD, the administration of TNBS to mice can release proinflammatory cytokines, followed by infiltration of T cell CD4^+^ phenotype. In these studies, the mice with colitis were orally administered with BAPs mostly with an amount of 50-500 mg/kg body weight/day for several days to weeks (Table 2). Then, the tissues are collected for common evaluation of anti-inflammation of BAPs using morphological, immunological, and biochemical assays [51], such as body weight, colonic length, disease activity index (DAI), lymphocyte proliferation, CD4^+^/CD8^+^ determination, secretory-immunoglobulin-A (s-IgA) measurement, immunoglobulin (IgA, IgM, and IgG) determination, and cytokine (IL-1, IL-2, IL-6, IL-8, IL-10, TNF-*α*, and IFN-*γ*) measurements (Table 2).

Numbers of BAPs derived from various food proteins (milk, plant, egg, soybean, meat, wheat, rice, potato, corn silk, fish, etc.) have been found to be well suited to treat inflammation or IBD symptoms *in vivo* (Table 2), such as Ala-Gln (AQ) [9, 52–54], DIKTNKPVIF [45], EWP [55], GLTSK [56], glycomacropeptide [57–60], lunasin [15], IRW [11, 61–63], IQW [62–64], KGHYAERVG [65], KPV [66], PTGADY [67], QCQCAVEGGL [68], QEPVL, QEPV [6], RILSILRHQNLLKELQDLAL [69], SSEDIKE [70], TMKLLLVTL [43], VPP [31, 46, 71, 72], IPP [71, 72], VPY [33], WH [41], casein hydrolysates [73], soybean dipeptides and tripeptides [74], peptide P-317 [75], pyroGlu-Leu [76], *β*-Casofensin [77], *γ*-EC, and *γ*-EV [47]. These studies suggest that oral administration of food-derived BAPs have anti-inflammatory effects, and they can be the therapeutic agents for inflammatory-related diseases, including IBD [78].

Oral administration of dipeptide AQ reduced inflammatory cytokine expression, enhancing the mucosa recovery in DSS-induced mice [53]. Likewise, intravenous infusion with AQ to calves with early weaned stress can increase concentrations of IgA, IgG, s-IgA, CD2^+^ and CD4^+^ lymphocytes, and CD4^+^/CD8^+^ ratio; therefore, the diarrhea occurrence was decreased [52]. Bean protein is also a rich resource for BAPs. For example, bean- and yeast extract-derived flavor peptide *γ*-EC and *γ*-EV can inhibit the inflammation in IBD mice [47]. Soybean-derived dipeptides and tripeptides decreased the colonic expressions of proinflammatory IFNG, IL-1B, IL-12B, TNF, and IL-17A and MPO activity and increased Foxp3 expression and CD4^+^CD25^+^ T cells; therefore, the colon and ileum inflammation of piglets with DSS-induced colitis was attenuated [74]. In addition, with the infusion of 150 mg/kg of egg white protein-derived EWP, weight loss, crypt distortion, IL-6 and TNF-*α* concentrations, and expressions of IL-1*β*, IL-8, IL-17, and IFN-*γ* in the colon of piglets with DSS-induced colitis can be reduced, and gut barrier function was restored [55], as well as the barrier protection effects of milk-derived *β*-Casofensin [77] and dipeptide AQ [53]. Therefore, food-derived BAPs can contribute to disease treatment through modifying intestinal barrier function [79].

In DSS-induced mice, antioxidant enzyme activities and microbial diversity and abundance were increased and the colitis was attenuated by egg white protein-derived IRW and IQW [63]. Oral administration of corn silk extract-derived TMKLLLVTL suppressed IKK*β* activity, I*κ*B phosphorylation, NF-*κ*B activity, and IL-1*β* production in LPS-induced inflammatory mice [43]. Drinking water with soybean-derived tripeptide VPY can reduce DAI, weight loss, MPO activity, and expressions of IL-1*β*, IL-6, IL-17, IFN-*γ*, and TNF-*α* in colitis mice [33], suggesting that VPY can treat IBD. In addition, sardine muscle hydrolysate-derived dipeptide WH can reduce DSS-induced colitis symptoms, colonic cytokine expression, MAPK and I*κ*B*α* activation, and IL-8 secretion in colitis mice, indicating that WH can inhibit intestinal inflammation [41]. Favor peptide *γ*-EC and *γ*-EV inhibited I*κ*B*α* and JNK activation and expressions of IL-1*β*, IL-6, IL-17, INF-*γ*, and TNF-*α* and increased IL-10 expression in IBD mice [47]. Moreover, tripeptide KPV reduced intestinal inflammation by decreasing IL-1*β*, IL-6, IL-12, and IFN-*γ* expressions and attenuated colitis via PepT1 [66].

Milk protein is a rich source for BAPs, which has potential beneficial effects to the gut of humans and animals [80, 81]. Milk casein-derived VPP and IPP are two famous BAPs with antihypertensive and anti-inflammatory activities. Proinflammatory IL-6 and IL-1*β* were reduced, and atherosclerosis was attenuated by oral administration of VPP and IPP [71]. Arterial dysfunction was attenuated by drinking water with VPP and IPP through increasing vasorelaxation and nitrite and nitrate and reducing pulse wave velocity and cardiac and renal damage [72]. It was reported that VPP attenuated inflammation via the MAPK-JNK pathway by reducing monocytes, macrophages, CD18, IL-6, and MCP-1 in adipose inflammatory mice [46]. Milk casein-derived QEPVL and QEPV reduced nitric oxide (NO) release, increased anti-inflammatory IL-4 and IL-10 production, and decreased productions of IFN-*γ* and TNF-*α* in LPS-induced mice [6]. Milk *κ*-casein-derived glycomacropeptide inhibited inflammation and attenuated colitis via normalizing the inflammatory cytokine and the NF-*κ*B and MAPK pathways in previous studies [57–60].

From these *in vivo* studies, the evidences that the intestinal inflammation can be attenuated by oral administration of food protein-derived BAPs have been presented. As many studies have been performed recently, large-scale human and animal trials are still lacking [2]. It has been reviewed that numbers of BAPs can be transported into the bloodstream of humans or animals to exert bioactivities [3, 81]. However, there is still limitation for such *in vivo* studies due to the possible degradation of BAPs by peptidases in the gut and plasma or insufficient absorption [82]. In the future, more studies of humans and animals are needed to evaluate the anti-inflammatory effects of BAPs, as well as the doses, times, and kinetics in the body.

## 7. Peptide Transporter PepT1

The peptide transporter 1 (PepT1) can transport small peptides from the intestine into the bloodstream of humans or animals [83–85], particularly di- and tripeptides, and its expression in intestinal epithelial cells is increased when the intestine is suffering from inflammation [86], indicating that PepT1 is a gateway to inflammatory response [87]. Similarly, PepT1 can transport various BAPs into intestinal epithelial cells to exert bioactivities [3, 81], such as IPAV [23], KPV [66], LKP, IQW [88], LSW [89], IWH, IW [90], and VPY [33].

It was reported that anti-inflammatory tripeptide KPV can attenuate intestinal inflammation associated with PepT1 expression, and KPV lost the anti-inflammatory function without PepT1 expression, suggesting that PepT1 mediates the anti-inflammation of KPV [66]. It was reported that soy protein-derived tripeptide VPY exerted anti-inflammatory activity in cells also through PepT1, which can transport VPY into cells [33]. In addition, pharmacological inhibition of PepT1 can counteract the inhibition of IL-8 expression mediated by peptide IPAV [23]. Moreover, the anti-inflammatory effect of meat-derived carnosine (*β*-Ala-His) was inhibited by dipeptide Gly-Sar, a PepT1 substrate [35]. These findings indicate that PepT1 is a promising target to treat intestinal inflammation by transporting sufficient short-chain BAPs into colonic cells [10]. In conclusion, PepT1 is a possible mechanism for the inhibition of intestinal inflammation by BAPs. However, this PepT1 pathway involved in anti-inflammation of BAPs still needs to be verified by further researches in the future (Figure 2).

## 8. Impact of Anti-Inflammatory Peptides on Gut Microbiota

When intestinal inflammation or IBD occurs, the gut microbial community would also change, such as the decrease of *Firmicutes* (particularly *Clostridium* groups) and the increase of *Bacteroides*, *Lactobacillus*, *Eubacterium*, and *Proteobacteria* [91]. In DSS-induced colitis mice, compositions and varieties of the gut microorganism (*Anaerotruncus*, *Bacteroides*, *Enterobacteriaceae*, *Lactobacilli*, and *Parabacteroides*) have changed [92]. In general, when defensins decline, the abundance of bacteria from *Bacteroides* and *Firmicutes* would be increased [93].

It was reported that BAPs can exert anti-inflammation via changing the gut microbiota in several studies [62, 63, 76]. For example, oral administration of anti-inflammatory peptide pyroGlu-Leu derived from wheat gluten can normalize the population of *Bacteroidetes* and *Firmicutes* in the colon of colitis mice [76]. Shannon and Simpson indices represent species richness and species evenness, respectively. The Simpson index and the abundance of *Coprococcus-1*, *Desulfovibrio*, and *Ruminococcaceae-UCG-014* were increased by tripeptides IRW and IQW. Additionally, IQW decreased the abundance of *Bacteroides* and increased *Parabacteroides*, while the levels of *Anaerotruncus*, *Ruminiclostridium-9*, and *Oscillibacter* were increased by IRW [63]. *Firmicutes* and *Actinobacteria* species were increased, and the proportions of *Bacteroidetes* and *Proteobacteria* species were decreased by oral administration of IRW and IQW; therefore, the colonic inflammation was inhibited via regulation of intestinal microorganisms [62]. In addition, dietary dipeptide GQ changed the gut microbiota beneficially through increasing alpha diversity, bacterial loading, abundance of anaerobes and fiber-degrading bacteria (*Phylum Fibrobacteres*), and short-chain fatty acids in the gut [94].

In conclusion, the gut microbiota is a promising mechanism for BAPs to inhibit intestinal inflammation. However, the information of the mechanism underlying the effects of BAPs on gut microbiota is still lacking, and it needs more studies to explore the interaction between anti-inflammation of BAPs and gut microbiota in the future.

## 9. Conclusions and Future Perspectives

In this review, the mechanism and pathways of food protein-derived BAPs to exert anti-inflammatory bioactivities were highlighted, including pathways (NF-*κ*B, MAPK, and JAK-STAT), PepT1, inflammatory mediators, and gut microbiota. Moreover, various *in vitro* and *in vivo* studies of BAPs on inflammation were reviewed, finding that PepT1 and gut microbiota are promising targets for the inhibition of BAPs on intestinal inflammation; however, their roles still need more further studies to be verified in the future.

The discovery of novel BAP sequences and their corresponding action mechanisms as well as gut microbiota and PepT1 involved in the mediation can provide new opportunities for better targeting of intestinal inflammation. More *in vivo* data, including pharmacokinetics and proper dosage and time of administration of BAPs, are needed before their application to humans and animals. The role of dietary BAPs in inhibiting intestinal inflammation represents a novel direction in nutraceuticals for people or animals with intestinal inflammation.

## Figures and Tables

**Table 1:** The *in vitro* effects of food-derived bioactive peptides on inhibiting inflammation.

Peptides	Origin	Object	Administration	Activities	Results	Reference
CR, FL, HC, LL, MK	Egg ovotransferrin	TNF-*α*-induced Caco-2 cells	0.05-2 mg/mL egg white digest	Reduce IL-8 secretion and expressions of TNF-*α*, IL-8, IL-6, IL-1*β*, and IL-12 and increase IL-10 expression	Inhibit intestinal inflammation	[17]
DEDTQAMPFR, DEDTQAMPF, MLGATSL, MSYSAGF	Egg white protein	TNF-*α*-induced Caco-2 cells	0.25 mg/mL peptide	Inhibit expressions of TNF-*α*, IL-8, IL-6, IL-1*β*, IL-12, JNK, I*κ*B, and p38 and increase IL-10 expression	Inhibit inflammation via the MAPK pathway	[18]
DYKKY	Milk whey protein	RAW 264.7 cells	10 and 100 *μ*g/mL	Inhibit expressions of IL-1*β*, COX-2, and TNF-*α* and productions of IL-1*β* and TNF-*α* and inhibit p38, p65, and I*κ*B*α* degradation	Inhibit inflammation via the NF-*κ*B pathway	[19]
EAMAPK, AVPYPQ	Milk casein	H_2_O_2_-induced IEC-6 cells	5-150 g/mL peptide	Reduce ROS levels and increase SOD and Nrf2 activities	Antioxidation	[13]
FLV	Soybean protein	TNF-*α*-induced RAW 264.7 and 3T3-L1 cells	0.1-1 *μ*M FLV	Inhibit productions of TNF-*α*, IL-6, and MCP-1 and expressions of JNK, IKK, and I*κ*B*α*	Inhibit inflammation	[20]
GPETAFLR	Lupine protein	THP-1-derived macrophages	100-500 *μ*g/mL GPETAFLR	Reduce expressions of TNF-*α*, IL-1*β*, and CCL2 and increase IL-10 expression	Prevent chronic inflammation	[21]
GPR	Amaranth protein	LPS-induced THP-1 and RAW 264.7 cells	1 mg/mL hydrolysate	Inhibit TNF-*α* secretion	Inhibit inflammation via the NF-*κ*B pathway	[22]
IPAV	Milk whey protein	TNF-*α*-induced Caco-2 cells	25-200 *μ*M IPAV	Reduce IL-8 and inhibit expressions of NF-*κ*B, ERK1/2, JNK1/2, Syk, and p38	Inhibit intestinal inflammation via PepT1	[23]
IRW	Egg ovotransferrin	TNF-*α*-induced human endothelial cells	50 *μ*M IRW	Inhibit ICAM-1, VCAM-1, MCP-1, and NF-*κ*B pathway	Inhibit vascular inflammation	[24]
IRW, IQW	Egg ovotransferrin	HUVECs	50 *μ*M IRW or IQW	Inhibit expressions of ICAM-1, VCAM-1, and NF-*κ*B pathway	Inhibit endothelial inflammation and oxidative stress	[12]
LDAVNR, MMLDF	Spirulina maxima	RBL-2H3 mast cells and EA.hy926 cells	200 *μ*M peptide	Reduce histamine release, IL-8 production, and ROS production	Inhibit inflammation	[25]
Lunasin	Defatted soybean meal protein	LPS-induced RAW 264.7 cells	100 *μ*M lunasin	Inhibit NO and PGE2 production and COX-2 and iNOS expressions	Inhibit inflammation	[14]
PAY	Salmon protein	LPS-induced RAW 264.7 cells	0.25-0.75 mM PAY	Reduce productions or expressions of NO, PGE2, TFN-*α*, IL-6, IL-1*β*, iNOS, and COX-2	Inhibit inflammation	[26]
PRRTRMMNGGR, MGPAMMRTMPG	Juice of cooked tuna	LPS-induced RAW 264.7 cells	100 *μ*g/mL hydrolysate	Inhibit secretions of IL-2, TNF-*α*, and IFN-*γ*	Inhibit inflammation	[27]
QCQQAVQSAV	Ruditapes philippinarum hydrolysate	LPS-induced RAW 264.7 cells	10-100 *μ*g/mL peptide	Inhibit NO production	Inhibit inflammation	[28]
QQQQQGGSQSQ, QEPQESQQ, QQQQQGGSQSQSQKG, PETMQQQQQQ	Germinated soybean protein	LPS-induced RAW 264.7 cells	2 mg/mL fraction	Inhibit NO and PGD2 production	Inhibit inflammation	[29]
SSEDIKE	Amaranth protein	Caco-2 cells	100-200 *μ*g/mL SSEDIKE	Reduce CCL20 and NF-*κ*B expressions	Inhibit inflammation	[30]
VPP	Milk casein	3T3-L1 adipocyte cells	1 mM VPP	Inhibit TNF-*α* expression	Inhibit inflammation via ACE-dependent cascade	[31]
VPP, IPP	Milk casein	3T3-F442A cells	50 *μ*M VPP or IPP	Upregulate PPAR*γ*, activate NF-*κ*B, and reduce adipokine	Inhibit inflammation	[32]
VPY	Soybean protein	Caco-2 and THP-1 cells	0.1-4 mM VPY	Inhibit IL-8 and TNF-*α* secretions	Treat IBD via PepT1	[33]
VH, LAN, IA, AL	Velvet antler protein from red deer	LPS-induced RAW 264.7 cells	100-500 *μ*g/mL peptide	Inhibit NO production	Inhibit inflammation	[34]
*β*-Ala-His	Meat products	H_2_O_2_-induced Caco-2 cells	—	Inhibit IL-8 and p38 and ERK activation	Inhibit inflammation via the MAPK and PepT1 pathways	[35]
pyroGlu-Leu	Wheat gluten	LPS-induced RAW 264.7 cells	200-800 *μ*g/mL peptide	Inhibit NO production, TNF-*α*, IL-6, and I*κ*B*α* degradation, and JNK, ERK, and p38 phosphorylation	Inhibit inflammation via the NF-*κ*B and MAPK pathways	[36]

3T3-L1: mouse preadipocytes; ACE: angiotensin-converting enzyme; Caco-2: human colorectal adenocarcinoma-derived intestinal epithelial cells; COX-2: cyclooxygenase-2; EA.hy926: human umbilical vein endothelial cells; PPAR*γ*: peroxisome proliferator-activated receptor gamma; RAW264.7: a mouse macrophage cell line; RAS: renin-angiotensin system; RBL-2H3: rat basophilic leukemia cells; ROS: reactive oxygen species; SOD: superoxide dismutase; THP-1: a human monocytic cell line; TNF-*α*: tumor necrosis factor *α*; HUVECs: human umbilical vein endothelial cells; ICAM-1: intercellular adhesion molecule-1; IL-1*β*: interleukin-1*β*; JNK: c-Jun N-terminal kinase; MAPK: mitogen-activated protein kinase; MCP-1: monocyte chemoattractant protein-1; NF-*κ*B: nuclear factor-*κ*B; NO: nitric oxide; VCAM-1: vascular cell adhesion molecule-1.

**Table 2:** The *in vivo* effect of bioactive peptides on inhibiting inflammation.

Peptides	Origin	Object	Administration	Activities	Results	Reference
AQ	Synthesis	Early-weaned calves	Intravenous infusion 1.01 g/kg BW/d AQ	Increase concentrations of CD2^+^ and CD4^+^ lymphocytes, CD4^+^/CD8^+^ ratio, and IgA, IgG, and s-IgA and improve intestinal integrity	Improve gain performance and decrease diarrhea occurrence	[52]
AQ	Synthesis	DSS-induced colitis C57BL/6 mice	Inject 75 mg/kg BW/d AQ	Reduce Th1/Th2/Th17, haptoglobin, IgG, chemokine, and MPO activity	Attenuate colitis	[9]
AQ	Synthesis	DSS-induced colitis C57BL/6 mice	Inject 75 mg/kg BW/d AQ	Increase colon length, TLR4, NF-*κ*B activation, and expressions of mucin 2, IL-17, and TNF-*α* and reduce IgG, DAI, and haptoglobin	Inhibit inflammation and enhance mucosa recovery	[53]
AQ	Synthesis	DSS-induced colitis C57BL/6 mice	Inject 75 mg/kg BW/d AQ	Reduce IL-17, Th17, and macrophage	Inhibit inflammation	[54]
DIKTNKPVIF	Potato protein hydrolysate	HFD-fed SAMP8 mice	Oral and intraperitoneal injection	Reduce expressions of p-p38, FGF-2, TNF-*α*, and IL-6	Attenuate proinflammatory reaction via the MAPK pathway	[45]
EWP	Egg white protein	DSS-induced IBD in piglets	Infuse 150 mg/kg BW EWP for 5 days	Reduce weight loss, crypt distortion, and expressions of TNF-*α*, IL-6, IL-1*β*, IFN-*γ*, IL-8, and IL-17 and restore gut barrier function	Manage IBD	[55]
GLTSK	Phaseolus vulgaris	AOM/DSS-induced colitis BALB/c mice	Oral 50 mg/kg BW/d GLTSK	Reduce DAI and neoplasms and enhance colon length	Attenuate colitis	[56]
Glycomacropeptide	Milk *κ*-casein	TNBS-induced ileitis rat	Oral 500 mg/kg BW/d peptide	Reduce DAI, MPO, alkaline phosphatase, iNOS, IL-1*β*, IL-17, and TNF	Attenuate ileitis via reducing IL-17	[57]
Glycomacropeptide	Milk *κ*-casein	DSS-induced colitis C57BL/6 female mice	Gavage 500 mg/kg BW/d peptide	Reduce DAI and normalize colonic expressions of IL-1*β*, IL17, IL23, IL6, TGF-*β*, IL10, and Foxp3	Inhibit inflammation	[58]
Glycomacropeptide	Milk *κ*-casein	DSS-induced colitis mice	Gavage 15 mg/d peptide	Increase BW and reduce DAI, CD4^+^, IFN-*γ*, and MPO activity	Inhibit colitis inflammation	[59]
Glycomacropeptide	Milk *κ*-casein	Oxazolone-induced ulcerative colitis BALB/c mice	Oral 50 mg/kg BW/d peptide	Inhibit NF-*κ*B and MAPK activations and reduce serum IL-1*β*, IL-5, IFN-*γ*, TNF-*α*, and IL-10 production	Attenuate colitis	[60]
Lunasin	Soybean protein	LPS-induced airway inflammation mice	Intranasal 20 *μ*g/mice lunasin	Reduce infiltration, goblet cell metaplasia, and Th2 cytokine expression	Alleviate inflammation	[15]
IRW	Egg ovotransferrin	Spontaneously hypertensive rat	Oral 15 mg/kg BW/d IRW	Reduce ICAM-1 and VCAM-1 expression	Inhibit inflammation and hypertension via the NF-*κ*B pathway	[11]
IRW	Egg ovotransferrin	LPS-induced inflammatory peritonitis in rat	Oral 40 mg/kg IRW in feed	Reduce serum TNF-*α* and IL-6 and MPO activity, increase Shannon index, and decrease Simpson indices	Attenuate inflammation	[61]
IRW, IQW	Egg ovotransferrin	DSS-induced colitis in mice	Drink water with 30 mg/mL peptide	Increase antioxidant enzyme activities and microbial diversity and abundance	Attenuate colitis	[63]
IRW, IQW	Egg ovotransferrin	DSS-induced colitis in mice	Oral 0.03% peptide in diet	Reduce TNF-*α* and IL-17	Inhibit colonic inflammation	[64]
IRW, IQW	Egg ovotransferrin	Citrobacter rodentium-induced colitis in mice	Oral 0.03% peptide in diet	Regulate intestinal microorganisms	Inhibit colonic inflammation	[62]
KGHYAERVG	Rice	Autoimmune encephalitis mice	Oral 100 mg/kg peptide	Reduce productions of IL-17, IFN-*γ*, IL-23, and IL-12 and increase T cells	Attenuate autoimmune encephalitis	[65]
KPV	C-terminal sequence of *α*-melanocyte stimulating hormone	DSS- and TNBS-induced colitis in mice	Drink water with 100 *μ*M KPV	Decrease expressions of IL-6, IL-12, IFN-*γ*, and IL-1*β*	Reduce intestinal inflammation via PepT1	[66]
PTGADY	Alaska pollock hydrolysates	Hydrogenated cortisone-treated mice	Oral 50-200 mg/kg BW/d hydrolysate	Increase productions of IL-2, IL-4, and IL-6	Immunomodulation	[67]
QCQCAVEGGL	Crassostrea gigas	DSS-induced colitis mice	Oral 50 mg/kg BW/d hydrolysate	Reduce IgE and increase spleen CD4^+^/CD8^+^	Attenuate colitis	[68]
QEPVL, QEPV	Milk casein	LPS-induced mice	Oral 200 mg/kg BW/d peptide	Reduce NO release, increase IL-4 and IL-10 production, and decrease IFN-*γ* and TNF-*α* production	Inhibit inflammation	[6]
RILSILRHQNLLKELQDLAL	Chromogranin A	DSS-induced colitis in mice	Intracolonic injection 2.5 mg/kg/day peptide	Reduce IL-18, active macrophages, increase TJ proteins	Attenuate colitis	[69]
SSEDIKE	Amaranth seeds	IgE-mediated food allergy mouse	Gavage 100 *μ*g SSEDIKE	Reduce productions of IgE, IgG, IL-5, IL-13, and NF-*κ*B and increase TGF-*β* and Foxp3 expressions	Inhibit intestinal inflammation	[70]
TMKLLLVTL	Corn silk extract	LPS-induced inflammatory mice	Oral 1 mg/kg peptide	Inhibit IL-*β*, IKK*β*, and I*κ*B phosphorylation and NF-*κ*B activation	Inhibit inflammation via the IKK*β*-NF-*κ*B pathways	[43]
VPP	Milk casein	HFD-induced adipose inflammation mice	Drink water with 0.3 mg/mL VPP for 10 weeks	Reduce monocytes, macrophages, CD18, IL-6, and MCP-1	Attenuate inflammation via the MAPK-JNK pathway	[46]
VPP	Milk casein	Obesity-induced adipose inflammation C57BL/6J mice	Drink water with 0.1% VPP for 4 months	Reduce TNF-*α* and IL-1*β* expression and macrophage accumulation and activation	Attenuate inflammation	[31]
VPP, IPP	Milk casein	Apolipoprotein E-deficient mice	Oral 60.2 or 125 *μ*mol/kg BW/d peptide	Reduce IL-6, IL-1*β*, and oxidized low-density lipoprotein receptor	Attenuate atherosclerosis	[71]
VPP, IPP	Milk *κ*-casein	L-NAME-treated rats	Drink water with 0.3 mg/mL VPP or IPP	Increase vasorelaxation and nitrite and nitrate and reduce cardiac and renal damage	Attenuate arterial dysfunction	[72]
VPY	Soybean protein	DSS-induced colitis BALB/C female mice	Drink water with 1 mg/mL VPY (100 mg/kg BW/d)	Reduce DAI, weight loss, and MPO activity and expressions of TNF-*α*, IL-6, IL-1*β*, IFN-*γ*, and IL-17	Treat IBD via PepT1	[33]
WH	Sardine muscle hydrolysate	DSS-induced colitis BALB/c mice	Oral 100 or 250 mg/kg BW/d WH for 14 d	Reduce DAI, cytokine expression, MAPK and I*κ*B*α* activation, and IL-8 secretion	Inhibit intestinal inflammation	[41]
Milk casein hydrolysates	Lactobacillus fermentation	TNBS-induced colitis mice	Oral 150 *μ*g/d hydrolysate	Reduce BW loss, microbial translocation, colonic DAI, and IFN-*γ* production	Treat colitis	[73]
Di- and tripeptides	Soybean protein	DSS-induced colitis pig	Infuse 250 mg/kg BW/d peptides	Reduce the expressions of IFNG, IL-1B, IL-12B, TNF, and IL-17A and MPO activity and increase Foxp3 expression and CD4^+^CD25^+^ T cells	Attenuate colon and ileum inflammation	[74]
Peptide P-317	Cyclic analog of morphiceptin	TNBS/DSS-induced colonic mice	Intraperitoneal 0.2 or oral 2 mg/kg BW/d peptide	Inhibit TNF-*α* and IL-1*β* expression and MPO activity	Treat IBD	[75]
pyroGlu-Leu	Wheat gluten	DSS-induced colitis mice	Gavage 0.01-10 mg/kg BW/d peptide	Reduce DAI and normalize colonic *Bacteroidetes* and *Firmicutes*	Treat IBD via gut microbiota	[76]
*β*-Casofensin	Milk protein	NMS-induced intestinal barrier alteration rat	Oral 10 *μ*L/kg BW/d peptide (0.01-100 *μ*M)	Reduce intestinal damages and prevent neonatal stress	Protect gut barrier	[77]
*γ*-EC, *γ*-EV	Beans and yeast extracts	DSS-induced BALB/C female mice	Gavage 50 or 150 mg/kg BW/d peptide	Inhibit I*κ*B*α* and JNK activation and the expressions of TNF-*α*, IL-6, INF-*γ*, IL-1*β*, and IL-17 and increase IL-10 expression	Inhibit colitis inflammation via the TNF-*α* pathway	[47]

ACE: angiotensin-converting enzyme; AOM: azoxymethane; BW: body weight; CD4^+^/CD8^+^: splenic T lymphocyte subpopulations; DAI: disease activity index; DSS: dextran sulfate sodium; Foxp3: a transcription factor that drives treg phenotypic differentiation; glycomacropeptide: a 64-amino acid peptide in stomach casein hydrolysis; HFD: high-fat diet; IBD: inflammatory bowel diseases; iNOS: inducible oxide nitric synthase; IFN: interferon; IKK*β*: inhibitory *κ*B kinase-*β*; IL-1*β*: interleukin-1*β*; KC: keratinocyte-derived chemokine; LPS: lipopolysaccharide; L-NAME: N(G)-nitro-L-arginine methyl ester hydrochloride; MCP-1: monocyte chemoattractant protein-1; MPO: myeloperoxidase; NF-*κ*B: nuclear factor-*κ*B; NMS: neonatal maternal separation; NO: nitric oxide; PPAR*γ*: peroxisome proliferator-activated receptor gamma; RAW264.7: a mouse macrophage cell line; SAMP8: senescence-accelerated mice prone 8; TGF-*β*: transforming growth factor *β*; TJ: tight junction; TLR4: toll-like receptor 4; Th1/2/17: T-helper-cell-associated cytokine 1/2/17; TNBS: 2,4,6-trinitrobenzene sulfonic acid.

## References

[1] Majumder K., Mine Y., Wu J. (2016). The potential of food protein-derived anti-inflammatory peptides against various chronic inflammatory diseases. *Journal of the Science of Food and Agriculture*.

[2] Chakrabarti S., Jahandideh F., Wu J. (2014). Food-derived bioactive peptides on inflammation and oxidative stress. *BioMed Research International*.

[3] Xu Q., Hong H., Wu J., Yan X. (2019). Bioavailability of bioactive peptides derived from food proteins across the intestinal epithelial membrane: a review. *Trends in Food Science and Technology*.

[4] de Silva S., Devlin S., Panaccione R. (2010). Optimizing the safety of biologic therapy for IBD. *Nature Reviews. Gastroenterology & Hepatology*.

[5] Bhat Z. F., Kumar S., Bhat H. F. (2017). Antihypertensive peptides of animal origin: a review. *Critical Reviews in Food Science and Nutrition*.

[6] Jiehui Z., Liuliu M., Haihong X. (2014). Immunomodulating effects of casein-derived peptides QEPVL and QEPV on lymphocytes in vitro and in vivo. *Food & Function*.

[7] Guha S., Majumder K. (2019). Structural-features of food-derived bioactive peptides with anti-inflammatory activity: a brief review. *Journal of Food Biochemistry*.

[8] Jurjus A. R., Khoury N. N., Reimund J.-M. (2004). Animal models of inflammatory bowel disease. *Journal of Pharmacological and Toxicological Methods*.

[9] Chu C.-C., Hou Y.-C., Pai M.-H., Chao C.-J., Yeh S.-L. (2012). Pretreatment with alanyl-glutamine suppresses T-helper-cell-associated cytokine expression and reduces inflammatory responses in mice with acute DSS-induced colitis. *The Journal of Nutritional Biochemistry*.

[10] Zhang H., Hu C. A. A., Kovacs-Nolan J., Mine Y. (2015). Bioactive dietary peptides and amino acids in inflammatory bowel disease. *Amino Acids*.

[11] Majumder K., Chakrabarti S., Morton J. S. (2013). Egg-derived tri-peptide IRW exerts antihypertensive effects in spontaneously hypertensive rats. *PLoS One*.

[12] Majumder K., Chakrabarti S., Davidge S. T., Wu J. (2013). Structure and activity study of egg protein ovotransferrin derived peptides (IRW and IQW) on endothelial inflammatory response and oxidative stress. *Journal of Agricultural and Food Chemistry*.

[13] Pepe G., Sommella E., Ventre G. (2016). Antioxidant peptides released from gastrointestinal digestion of “Stracchino” soft cheese: characterization, in vitro intestinal protection and bioavailability. *Journal of Functional Foods*.

[14] Dia V. P., Wang W., Oh V. L., Lumen B. O., de Mejia E. G. (2009). Isolation, purification and characterisation of lunasin from defatted soybean flour and in vitro evaluation of its anti-inflammatory activity. *Food Chemistry*.

[15] Yang X., Zhu J., Tung C.-Y. (2015). Lunasin alleviates allergic airway inflammation while increases antigen-specific tregs. *PLoS One*.

[16] Xu Q., Singh N., Hong H. (2019). Hen protein-derived peptides as the blockers of human bitter taste receptors T2R4, T2R7 and T2R14. *Food Chemistry*.

[17] Wang X., Zhao Y., Yao Y. (2017). Anti-inflammatory activity of di-peptides derived from ovotransferrin by simulated peptide-cut in TNF-*α*-induced Caco-2 cells. *Journal of Functional Foods*.

[18] Zhang M., Zhao Y., Yao Y. (2019). Isolation and identification of peptides from simulated gastrointestinal digestion of preserved egg white and their anti-inflammatory activity in TNF-*α*-induced Caco-2 cells. *The Journal of Nutritional Biochemistry*.

[19] Ma Y., Liu J., Shi H., Yu L. (2016). Isolation and characterization of anti-inflammatory peptides derived from whey protein. *Journal of Dairy Science*.

[20] Kwak S.-J., Kim C.-S., Choi M.-S. (2016). The soy peptide Phe–Leu–Val reduces TNF*α*-induced inflammatory response and insulin resistance in adipocytes. *Journal of Medicinal Food*.

[21] del Carmen Millán-Linares M., Millán F., Pedroche J., del Mar Yust M. (2015). GPETAFLR: a new anti-inflammatory peptide from *Lupinus angustifolius* L. protein hydrolysate. *Journal of Functional Foods*.

[22] Montoya-Rodríguez A., de Mejía E. G., Dia V. P., Reyes-Moreno C., Milán-Carrillo J. (2014). Extrusion improved the anti-inflammatory effect of amaranth (*Amaranthus hypochondriacus*) hydrolysates in LPS-induced human THP-1 macrophage-like and mouse RAW 264.7 macrophages by preventing activation of NF-*κ*B signaling. *Molecular Nutrition & Food Research*.

[23] Oyama M., Van Hung T., Yoda K., He F., Suzuki T. (2017). A novel whey tetrapeptide IPAV reduces interleukin-8 production induced by TNF-*α* in human intestinal Caco-2 cells. *Journal of Functional Foods*.

[24] Huang W., Chakrabarti S., Majumder K., Jiang Y., Davidge S. T., Wu J. (2010). Egg-derived peptide IRW inhibits TNF-*α*-induced inflammatory response and oxidative stress in endothelial cells. *Journal of Agricultural and Food Chemistry*.

[25] Vo T.-S., Ryu B., Kim S.-K. (2013). Purification of novel anti-inflammatory peptides from enzymatic hydrolysate of the edible microalgal Spirulina maxima. *Journal of Functional Foods*.

[26] Ahn C. B., Cho Y. S., Je J. Y. (2015). Purification and anti-inflammatory action of tripeptide from salmon pectoral fin byproduct protein hydrolysate. *Food Chemistry*.

[27] Cheng M.-L., Wang H.-C., Hsu K.-C., Hwang J.-S. (2015). Anti-inflammatory peptides from enzymatic hydrolysates of tuna cooking juice. *Food and Agricultural Immunology*.

[28] Lee S.-J., Kim E.-K., Kim Y.-S. (2012). Purification and characterization of a nitric oxide inhibitory peptide from *Ruditapes philippinarum*. *Food and Chemical Toxicology*.

[29] González-Montoya M., Hernández-Ledesma B., Silván J. M., Mora-Escobedo R., Martínez-Villaluenga C. (2018). Peptides derived from in vitro gastrointestinal digestion of germinated soybean proteins inhibit human colon cancer cells proliferation and inflammation. *Food Chemistry*.

[30] Moronta J., Smaldini P. L., Docena G. H., Añón M. C. (2016). Peptides of amaranth were targeted as containing sequences with potential anti-inflammatory properties. *Journal of Functional Foods*.

[31] Sawada Y., Sakamoto Y., Toh M. (2015). Milk-derived peptide Val-Pro-Pro (VPP) inhibits obesity-induced adipose inflammation via an angiotensin-converting enzyme (ACE) dependent cascade. *Molecular Nutrition & Food Research*.

[32] Chakrabarti S., Wu J. (2015). Milk-derived tripeptides IPP (Ile-Pro-Pro) and VPP (Val-Pro-Pro) promote adipocyte differentiation and inhibit inflammation in 3T3-F442A cells. *PLoS One*.

[33] Kovacs-Nolan J., Zhang H., Ibuki M. (2012). The PepT1-transportable soy tripeptide VPY reduces intestinal inflammation. *Biochimica et Biophysica Acta (BBA) - General Subjects*.

[34] Zhao L., Wang X., Zhang X.-L., Xie Q.-F. (2016). Purification and identification of anti-inflammatory peptides derived from simulated gastrointestinal digests of velvet antler protein ( Cervus elaphus Linnaeus). *Journal of Food and Drug Analysis*.

[35] Son D. O., Satsu H., Kiso Y., Totsuka M., Shimizu M. (2008). Inhibitory effect of carnosine on interleukin-8 production in intestinal epithelial cells through translational regulation. *Cytokine*.

[36] Hirai S., Horii S., Matsuzaki Y. (2014). Anti-inflammatory effect of pyroglutamyl-leucine on lipopolysaccharide-stimulated RAW 264.7 macrophages. *Life Sciences*.

[37] Li S., Liu L., He G., Wu J. (2018). Molecular targets and mechanisms of bioactive peptides against metabolic syndromes. *Food & Function*.

[38] Li S., Bu T., Zheng J., Liu L., He G., Wu J. (2019). Preparation, bioavailability, and mechanism of emerging activities of Ile-Pro-Pro and Val-Pro-Pro. *Comprehensive Reviews in Food Science and Food Safety*.

[39] Zhang T., McCarthy J., Wang G., Liu Y., Guo M. (2015). Physiochemical properties, microstructure, and probiotic survivability of nonfat goats’ milk yogurt using heat-treated whey protein concentrate as fat replacer. *Journal of Food Science*.

[40] Tanaka M., Hong S. M., Akiyama S., Hu Q. Q., Matsui T. (2015). Visualized absorption of anti-atherosclerotic dipeptide, Trp-His, in Sprague-Dawley rats by LC-MS and MALDI-MS imaging analyses. *Molecular Nutrition & Food Research*.

[41] Kobayashi Y., Kovacs-Nolan J., Matsui T., Mine Y. (2015). The anti-atherosclerotic dipeptide, Trp-His, reduces intestinal inflammation through the blockade of L-type Ca^2+^ channels. *Journal of Agricultural and Food Chemistry*.

[42] Tak P. P., Firestein G. S. (2001). NF-*κ*B: a key role in inflammatory diseases. *The Journal of Clinical Investigation*.

[43] Ho T.-Y., Li C.-C., Lo H.-Y., Chen F.-Y., Hsiang C.-Y. (2017). Corn silk extract and its bioactive peptide ameliorated lipopolysaccharide-induced inflammation in mice via the nuclear factor-*κ*B signaling pathway. *Journal of Agricultural and Food Chemistry*.

[44] Roy P. K., Rashid F., Bragg J. (2008). Role of the JNK signal transduction pathway in inflammatory bowel disease. *World Journal of Gastroenterology*.

[45] Dumeus S., Shibu M. A., Lin W.-T. (2018). Bioactive peptide improves diet-induced hepatic fat deposition and hepatocyte proinflammatory response in SAMP8 ageing mice. *Cellular Physiology and Biochemistry*.

[46] Aihara K., Osaka M., Yoshida M. (2014). Oral administration of the milk casein-derived tripeptide Val-Pro-Pro attenuates high-fat diet-induced adipose tissue inflammation in mice. *The British Journal of Nutrition*.

[47] Zhang H., Kovacs-Nolan J., Kodera T., Eto Y., Mine Y. (2015). *γ*-Glutamyl cysteine and *γ*-glutamyl valine inhibit TNF-*α* signaling in intestinal epithelial cells and reduce inflammation in a mouse model of colitis via allosteric activation of the calcium-sensing receptor. *Biochimica et Biophysica Acta (BBA) - Molecular Basis of Disease*.

[48] Chalamaiah M., Yu W., Wu J. (2018). Immunomodulatory and anticancer protein hydrolysates (peptides) from food proteins: a review. *Food Chemistry*.

[49] Chatterton D. E. W., Nguyen D. N., Bering S. B., Sangild P. T. (2013). Anti-inflammatory mechanisms of bioactive milk proteins in the intestine of newborns. *The International Journal of Biochemistry & Cell Biology*.

[50] Aihara K., Ishii H., Yoshida M. (2009). Casein-derived tripeptide, Val-Pro-Pro (VPP), modulates monocyte adhesion to vascular endothelium. *Journal of Atherosclerosis and Thrombosis*.

[51] Maestri E., Marmiroli M., Marmiroli N. (2016). Bioactive peptides in plant-derived foodstuffs. *Journal of Proteomics*.

[52] Zhou Y., Zhang P., Deng G., Liu X., Lu D. (2012). Improvements of immune status, intestinal integrity and gain performance in the early-weaned calves parenterally supplemented with l-alanyl-l-glutamine dipeptide. *Veterinary Immunology and Immunopathology*.

[53] Hou Y.-C., Chu C.-C., Ko T.-L., Yeh C.-L., Yeh S.-L. (2013). Effects of alanyl-glutamine dipeptide on the expression of colon-inflammatory mediators during the recovery phase of colitis induced by dextran sulfate sodium. *European Journal of Nutrition*.

[54] Hou Y.-C., Liu J.-J., Pai M.-H., Tsou S.-S., Yeh S.-L. (2013). Alanyl-glutamine administration suppresses Th17 and reduces inflammatory reaction in dextran sulfate sodium-induced acute colitis. *International Immunopharmacology*.

[55] Lee M., Kovacs-Nolan J., Archbold T. (2009). Therapeutic potential of hen egg white peptides for the treatment of intestinal inflammation. *Journal of Functional Foods*.

[56] Luna-Vital D. A., González de Mejía E., Loarca-Piña G. (2017). Dietary peptides from phaseolus vulgaris L. reduced AOM/DSS-induced colitis-associated colon carcinogenesis in Balb/c mice. *Plant Foods for Human Nutrition*.

[57] Requena P., Daddaoua A., Martínez-Plata E. (2008). Bovine glycomacropeptide ameliorates experimental rat ileitis by mechanisms involving downregulation of interleukin 17. *British Journal of Pharmacology*.

[58] López-Posadas R., Requena P., González R. (2010). Bovine glycomacropeptide has intestinal antiinflammatory effects in rats with dextran sulfate-induced colitis. *The Journal of Nutrition*.

[59] Ortega-González M., Capitán-Cañadas F., Requena P. (2014). Validation of bovine glycomacropeptide as an intestinal anti-inflammatory nutraceutical in the lymphocyte-transfer model of colitis. *The British Journal of Nutrition*.

[60] Ming Z., Jia Y., Yan Y., Pang G., Chen Q. (2015). Amelioration effect of bovine casein glycomacropeptide on ulcerative colitis in mice. *Food and Agricultural Immunology*.

[61] Jiao H., Zhang Q., Lin Y., Gao Y., Zhang P. (2019). The ovotransferrin-derived peptide IRW attenuates lipopolysaccharide-induced inflammatory responses. *BioMed Research International*.

[62] Ma Y., Ding S., Liu G. (2019). Egg protein transferrin-derived peptides IRW and IQW regulate citrobacter rodentium-induced, inflammation-related microbial and metabolomic profiles. *Frontiers in Microbiology*.

[63] Liu G., Yan W., Ding S. (2018). Effects of IRW and IQW on oxidative stress and gut microbiota in dextran sodium sulfate-induced colitis. *Cellular Physiology and Biochemistry*.

[64] Ma Y., Jiang H., Fang J., Liu G. (2019). IRW and IQW reduce colitis-associated cancer risk by alleviating DSS-induced colonic inflammation. *BioMed Research International*.

[65] Shapira E., Brodsky B., Proscura E., Nyska A., Erlanger-Rosengarten A., Wormser U. (2010). Amelioration of experimental autoimmune encephalitis by novel peptides: involvement of T regulatory cells. *Journal of Autoimmunity*.

[66] Dalmasso G., Charrier–Hisamuddin L., Thu Nguyen H. T., Yan Y., Sitaraman S., Merlin D. (2008). PepT1-mediated tripeptide KPV uptake reduces intestinal inflammation. *Gastroenterology*.

[67] Hou H., Fan Y., Wang S., Si L., Li B. (2016). Immunomodulatory activity of Alaska pollock hydrolysates obtained by glutamic acid biosensor – artificial neural network and the identification of its active central fragment. *Journal of Functional Foods*.

[68] Hwang J.-W., Lee S.-J., Kim Y.-S. (2012). Purification and characterization of a novel peptide with inhibitory effects on colitis induced mice by dextran sulfate sodium from enzymatic hydrolysates of *Crassostrea gigas*. *Fish & Shellfish Immunology*.

[69] Eissa N., Hussein H., Kermarrec L. (2017). Chromofungin ameliorates the progression of colitis by regulating alternatively activated macrophages. *Frontiers in Immunology*.

[70] Moronta J., Smaldini P. L., Fossati C. A., Añon M. C., Docena G. H. (2016). The anti-inflammatory SSEDIKE peptide from Amaranth seeds modulates IgE-mediated food allergy. *Journal of Functional Foods*.

[71] Nakamura T., Hirota T., Mizushima K. (2013). Milk-derived peptides, Val-Pro-Pro and Ile-Pro-Pro, attenuate atherosclerosis development in apolipoprotein E–deficient mice: a preliminary study. *Journal of Medicinal Food*.

[72] Nonaka A., Nakamura T., Hirota T. (2014). The milk-derived peptides Val-Pro-Pro and Ile-Pro-Pro attenuate arterial dysfunction in L-NAME-treated rats. *Hypertension Research*.

[73] Espeche Turbay M. B., de Moreno de LeBlanc A., Perdigón G., Savoy de Giori G., Hebert E. M. (2012). *β*-Casein hydrolysate generated by the cell envelope-associated proteinase of Lactobacillus delbrueckii ssp. lactis CRL 581 protects against trinitrobenzene sulfonic acid-induced colitis in mice. *Journal of Dairy Science*.

[74] Young D., Ibuki M., Nakamori T., Fan M., Mine Y. (2012). Soy-derived di- and tripeptides alleviate colon and ileum inflammation in pigs with dextran sodium sulfate-induced colitis. *The Journal of Nutrition*.

[75] Sobczak M., Zakrzewski P. K., Cygankiewicz A. I. (2014). Anti-inflammatory action of a novel orally available peptide 317 in mouse models of inflammatory bowel diseases. *Pharmacological Reports*.

[76] Wada S., Sato K., Ohta R. (2013). Ingestion of low dose pyroglutamyl leucine improves dextran sulfate sodium-induced colitis and intestinal microbiota in mice. *Journal of Agricultural and Food Chemistry*.

[77] Bessette C., Henry G., Sekkal S. (2016). Oral administration of a casein matrix containing *β*-casofensin protects the intestinal barrier in two preclinical models of gut diseases. *Journal of Functional Foods*.

[78] La Manna S., Di Natale C., Florio D., Marasco D. (2018). Peptides as therapeutic agents for inflammatory-related diseases. *International Journal of Molecular Sciences*.

[79] Martínez-Augustin O., Rivero-Gutiérrez B., Mascaraque C., Sánchez de Medina F. (2014). Food derived bioactive peptides and intestinal barrier function. *International Journal of Molecular Sciences*.

[80] Xu Q. B., Zhang Y. D., Zheng N. (2020). Short communication: decrease of lipid profiles in cow milk by ultra-high-temperature treatment but not by pasteurization. *Journal of Dairy Science*.

[81] Xu Q., Yan X., Zhang Y., Wu J. (2019). Current understanding of transport and bioavailability of bioactive peptides derived from dairy proteins: a review. *International Journal of Food Science and Technology*.

[82] Santiago-Lopez L., Gonzalez-Cordova A. F., Hernandez-Mendoza A., Vallejo-Cordoba B. (2017). Potential use of food protein-derived peptides in the treatment of inflammatory diseases. *Protein & Peptide Letters*.

[83] Xu Q., Liu Z., Liu H. (2018). Functional characterization of oligopeptide transporter 1 of dairy cows. *Journal of Animal Science and Biotechnology*.

[84] Xu Q., Wu Y., Liu H., Xie Y., Huang X., Liu J. (2014). Establishment and characterization of an omasal epithelial cell model derived from dairy calves for the study of small peptide absorption. *PLoS One*.

[85] Xu Q., Liu H., Zhao F. (2018). Mechanism of peptide absorption in the isolated forestomach epithelial cells of dairy cows. *Journal of the Science of Food and Agriculture*.

[86] Ingersoll S. A., Ayyadurai S., Charania M. A., Laroui H., Yan Y., Merlin D. (2012). The role and pathophysiological relevance of membrane transporter PepT1 in intestinal inflammation and inflammatory bowel disease. *American Journal of Physiology-Gastrointestinal and Liver Physiology*.

[87] Charrier L., Merlin D. (2006). The oligopeptide transporter hPepT1: gateway to the innate immune response. *Laboratory Investigation*.

[88] Xu Q., Fan H., Yu W., Hong H., Wu J. (2017). Transport study of egg-derived antihypertensive peptides (LKP and IQW) using Caco-2 and HT29 coculture monolayers. *Journal of Agricultural and Food Chemistry*.

[89] Lin Q., Xu Q., Bai J., Wu W., Hong H., Wu J. (2017). Transport of soybean protein-derived antihypertensive peptide LSW across Caco-2 monolayers. *Journal of Functional Foods*.

[90] Fan H., Xu Q., Hong H., Wu J. (2018). Stability and transport of spent hen-derived ACE-inhibitory peptides IWHHT, IWH, and IW in human intestinal Caco-2 cell monolayers. *Journal of Agricultural and Food Chemistry*.

[91] Nagalingam N. A., Lynch S. V. (2012). Role of the microbiota in inflammatory bowel diseases. *Inflammatory Bowel Diseases*.

[92] Håkansson Å., Tormo-Badia N., Baridi A. (2015). Immunological alteration and changes of gut microbiota after dextran sulfate sodium (DSS) administration in mice. *Clinical and Experimental Medicine*.

[93] Salzman N. H., Hung K., Haribhai D. (2010). Enteric defensins are essential regulators of intestinal microbial ecology. *Nature Immunology*.

[94] Yan Y., Xu B., Yin B. (2020). Modulation of gut microbial community and metabolism by dietary glycyl-glutamine supplementation may favor weaning transition in piglets. *Frontiers in Microbiology*.

